# Mitochondria, Cognitive Impairment, and Alzheimer's Disease

**DOI:** 10.4061/2009/951548

**Published:** 2009-07-06

**Authors:** M. Mancuso, V. Calsolaro, D. Orsucci, C. Carlesi, A. Choub, S. Piazza, G. Siciliano

**Affiliations:** Department of Neuroscience, Neurological Clinic, University of Pisa, Via Roma 67, 56126 Pisa, Italy

## Abstract

To date, the beta amyloid (A*β*) cascade hypothesis remains the main pathogenetic model of Alzheimer's disease (AD), but its role in the majority of sporadic AD cases is unclear. The “mitochondrial cascade hypothesis” could explain many of the biochemical, genetic, and pathological features of sporadic AD. Somatic mutations in mitochondrial DNA (mtDNA) could cause energy failure, increased oxidative stress, and accumulation of A*β*, which in a vicious cycle reinforce the mtDNA damage and the oxidative stress. Despite the evidence of mitochondrial dysfunction in AD, no causative mutations in the mtDNA have been detected so far. Indeed, results of studies on the role of mtDNA haplogroups in AD are controversial. In this review we discuss the role of the mitochondria, and especially of the mtDNA, in the cascade of events leading to neurodegeneration, dementia, and AD.

## 1. Introduction

Mitochondria are dynamic and pleomorphic organelles, composed of a smooth outer membrane surrounding an inner membrane of significantly larger surface area that, in turn, surrounds a protein-rich core, the matrix. They contain 2 to 10 molecules of DNA, the mitochondrial DNA (mtDNA) [1]. Most likely, mitochondria are derived from aerobic prokaryotes integrated into nucleated cells. Mitochondria are ubiquitous in eukaryotes. Their number per cell ranges from zero in erythrocytes to ten thousands in striated muscle cells. Their main function is to support aerobic respiration and to provide energy as adenosine triphosphate (ATP), by means of the electron transport chain (ETC). The ETC is needed for oxidative phosphorilation (which provides the cell with the most efficient energetic outcome in terms of ATP production), and consists of four multimeric protein complexes located in the inner mitochondrial membrane [1]. The ETC also requires cytocrome *c * (cyt *c*) and a small electron carrier, coenzyme Q10 (CoQ10, or ubiquinone). Electrons are transported along the complexes to molecular oxygen (O_2_), finally producing water. At the same time, protons are pumped across the mitochondrial inner membrane, from the matrix to the intermembrane space, by complexes I, III, and IV. This process creates an electrochemical proton gradient. ATP is produced by the influx of these protons back through the complex V, or ATP synthase (the “rotary motor”) [2]. This metabolic pathway is under control of both nuclear (nDNA) and mitochondrial genomes [1, 3]. Other functions concern mediation of cell death by apoptosis, heat production by decoupling of the oxidative phosphorylation, translation and transcription of mitochondrial genes [4]. In mitochondria, parts of some metabolic processes take place, such as *β*-oxidation, citrate acid cycle, degradation of aminoacids, haem biosynthesis, steroid metabolism, uric acid cycle, and others.

The mtDNA is a 16.5-kb circular minichromosome built up of complementary H- and L-strands [1]. The mitochondrial genome contains 37 genes, 13 of which encode for subunits of ETC complexes, 22 for transfer RNAs (tRNAs), and two for ribosomal RNAs (rRNAs) [1]. The 13 mtDNA-encoded polypeptides are part of the respiratory system and are assembled together with nuclear-encoded subunits. Seven of them belong to complex I or NADH dehydrogenase, NADH:ubiquinone oxidoreductase (ND1, ND2, ND3, ND4, ND4L, ND5, ND6), one to complex III or ubiquinol: ferricytochrome *c*-oxidoreductase, three to complex IV or cyt *c * oxidase (COX I, COX II, and COX III) and two to complex V or ATP synthase (ATPase6 and ATPase8). The remaining mitochondrial proteins, including all the subunits of complex II, are encoded by nDNA. mtDNA is not protected by histones, its mutation rate is 10 times higher than that of nDNA, and it does not undergo recombination during meiosis [5]. The mitochondrial genome is maternally inherited.

Mitochondrial diseases (MD) are a group of disorders caused by impairment of the mitochondrial ETC. The genetic classification of MD distinguishes disorders due to defects in mtDNA from those due to defects in nDNA [3]. MtDNA mutations are characterized by some peculiarities compared to nDNA mutations, and can occur spontaneously or be inherited from the mother. MtDNA mutations are classified as either large-scale rearrangements (partial deletions or duplications), usually sporadic, or point mutations, which are usually maternally inherited, and concern genes responsible for protein synthesis (rRNAs or tRNAs), or genes encoding subunits of the ETC [3]. The phenotypic expression of mtDNA mutations depends on the affected gene, its tissue distribution, and the different dependency of different organs and tissues on the mitochondrial energy supply. If the load of mutant mitochondrial genomes exceeds a certain amount in a given tissue, the effect of the mutation can be no longer compensated by wild-type mtDNA (“threshold effect”). Organs and tissues that predominantly rely on mitochondrial aerobic energy production, such as visual and auditory pathways, heart, central nervous system (CNS), and skeletal muscle, are more frequently involved [1]. Phenotype expression often require the influence of various factors, such as nuclear modifier genes, environmental influence factors, or the presence of mtDNA polymorphisms. Clusters of mtDNA variants act as predisposing haplotypes increasing the risk of disease [6].

**Figure 1:**
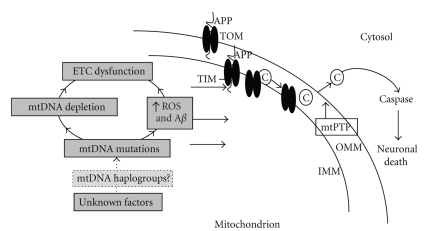
*A proposed mechanism of mitochondrial induced cell death in Alzheimer's disease*. Legend: A*β*; amyloid-*β*; ROS reactive oxygen species; ETC electron transport chain; mtPTP mitochondrial permeability transition pore; C cytochrome *c *; IMM inner mitochondrial membrane; OMM outer mitochondrial membrane; APP amyloid precursor protein; TOM and TIM protein importation translocases of the mitochondrial outer and inner membranes. For further details, see text. (*Modified from Mancuso M et al., Antioxid Redox Signal 2007;9:1631–1646*).

Mitochondria play a central role in apoptotic cell death, and mitochondrial dysfunction appears to have a certain impact on the pathogenesis of several neurodegenerative diseases, such as A**l**zheimer's Disease (AD) [7]. In the past twenty years research has been directed at clarifying the involvement of mitochondria and defects in mitochondrial oxidative phosphorylation in late-onset neurodegenerative disorders. Morphological, biochemical, and genetic abnormalities of the mitochondria in several AD tissues have been reported. Impaired mitochondrial respiration, particularly COX (complex V) deficiency, has been observed in brain, platelets, and fibroblasts of AD patients [7]. The “mitochondrial cascade hypothesis” [8] could explain many of the biochemical, genetic, and pathological features of sporadic AD. Somatic mutations in mtDNA could cause energy failure, increased oxidative stress, and accumulation of A*β*, which in a vicious cycle reinforces mtDNA damage and oxidative stress (Figure 1). Despite the evidence of mitochondrial dysfunction in AD, and despite the cognitive impairment frequently reported in patients with mtDNA mutation, no causative mutations in the mtDNA have been linked to AD so far. Indeed, results of studies on the role of mtDNA haplogroups in AD are controversial. Here, we discuss the role of the mitochondria, and especially of the mtDNA, in the cascade of events leading to AD, after briefly reviewing the cognitive alterations present in patients harboring mtDNA mutations.

## 2. Cognitive Impairment in Mitochondrial Disorders

MD may affect the only muscular tissue or present as a multisystem disease [1]. One of the most frequently affected organs in mitochondrial diseases, in addition to the skeletal muscle, is CNS, with a lot of different possible manifestations, such as epilepsy, stroke-like episodes, ataxia, spasticity, and dementia. MD associated with cognitive impairment include mitochondrial encephalomyopathy, lactic acidosis, and stroke-like episodes syndrome (MELAS), Kearns-Sayre syndrome (KSS), Leigh syndrome, and many others [9]. At the onset, cognitive impairment may be partial, appearing with specific cognitive deficits, particularly in abstract reasoning, verbal memory, visual memory, language (naming and fluency), executive or constructive functions, calculation, attention (attention deficit disorder and decreased attention span), or visuospatial functions [9–12]. Cognitive functions and intellectual abilities may decline from initially focal cognitive impairment to dementia [9–17]. Dementia is defined as chronic and disabling memory impairment, with involvement of at least one other cognitive function, resulting in reduced competence to judge or to reflect [18]. Cognitive functions that can be involved are memory for verbal and nonverbal materials, language, orientation, constructional abilities, abstract thinking, problem solving, or praxis. Changes in personality are often associated with dementia, and also behavioral alteration may occur during the evolution of the cognitive decline [19]. Diagnosis of mitochondrial dementia requires neuropsychological testing, cerebrospinal fluid investigations, visually-evoked potentials, EEG, brain CT and MRI scans, phosphorus or proton-magnetic resonance spectroscopy, SPECT, or positron emission tomography with fluorine 18-labeled deoxyglucose (FDG-PET) [18]. High-resolution regional cerebral blood flow obtained through Tc-99m ethylcysteinate dimer SPECT can better localize and assess the extent of brain damage in patients with suspected MD and only subtle changes on MRI [20]. Recently, reduced regional glucose metabolism has been observed in the frontotemporal region of two siblings with mtDNA multiple deletions and a Mitochondrial neurogastrointestinal encephalomyopathy (MNGIE)-like disorder, by means of FDG-PET [21]. The discrepancy between the absence of clinical and MRI signs of cerebral involvement and the substantial impairment of glucose metabolism could reflect a chronic subclinical encephalopathy [21].

In conclusion, pathogenic mutations in the mtDNA have been found in patients with cognitive disorders. However, little is known about whether pathogenic mtDNA mutations and the resultant mitochondrial respiration deficiencies contribute to the expression of cognitive alterations, such as impairments of learning and memory. Recently Tanaka et al. [22] used two groups of transmitochondrial mice (mito-mice) with heteroplasmy for wild-type and pathogenically deleted mtDNA. The “low" group carried 50% or less of deleted mtDNA, whilst the “high" group carried more than 50% of deleted mtDNA [22]. These authors observed that deleted mtDNA load did not affect learning and temporal memory, whereas the “high” group showed severe impairment of retention and consolidation of memory trace [22]. In the visual cortex and dentate gyrus of these mice have been reported respiratory system deficiencies, in particular of COX activity [22]. Therefore, high loads of pathogenically mutated mtDNA may be responsible for COX deficiency and for the preferential impairment of remote memory.

Despite the cognitive impairment frequently reported in MD patients, and despite the high prevalence of AD, AD patients harbouring a mtDNA mutations have never been reported.

## 3. Mitochondrial Dysfunction and Alzheimer's Disease

AD is the most common form of dementia in the elderly. It is clinically characterized by impairment of cognitive functions and changes in behavior and personality. AD is associated with progressive and irreversible loss of neurons, particularly in the cortex and hippocampus, extracellular senile plaques containing aggregated A*β*, and neurofibrillary tangles composed of the hyperphosphorylated form of the microtubular protein tau [23]. The A*β* cascade hypothesis remains the main pathogenetic model of familial AD with mutation in amyloid precursor protein (APP) and presenilin genes [24], but its role in the majority of sporadic AD cases without mutations in these genes (accounting for *the great majority * of AD cases) is still unclear. The A*β* peptide is the result of a regulated intramembrane proteolysis of APP by the sequential cleavage by *β*- and *γ*-secretases [25, 26]. A*β* plaques might be the cause of toxicity, loss of synapses, and ultimately neuronal death [27, 28]. The exact mechanisms of the neurotoxicity of A*β* are still unknown. Several lines of evidence suggest that A*β* exerts its toxicity intracellularly [29, 30], pointing to a role of the mitochondrion in this process [31].

Mitochondrial dysfunction is a prominent feature of AD, but the underlying mechanism is still unclear. Mitochondrial A*β* accumulation impairs neuronal function contributing to cellular dysfunction in a transgenic APP mouse model [32]. During the early stages of AD a reduced number of mitochondria in neurons has been reported [33], as well as decreased brain glucose metabolism [34]. Moreover, reduced activities of both tricarboxylic acid cycle enzymes [35] and COX [36, 37] have been reported .

Because A*β* is not produced locally in the mitochondrion [38, 39], Hansson Petersen et al. [40] recently investigated the mechanism by which A*β* is taken up by mitochondria. The most important system providing the translocation of A*β* precursors with mitochondrial target signals involves the translocase of the outer membrane (TOM) and the translocase of the inner membrane (TIM). Targeting signals are first recognized by TOM receptors (Tom20, Tom22, and Tom70), and then traslocated by Tom40, the general import pore of TOM [41, 42]. Subsequently, A*β* precursors are directed to the matrix via the Tim23 complex [42].

In isolated rat mitochondria has been observed that A*β* is imported into mitochondria via the TOM complex [40]. Preincubating mitochondria with antibodies directed toward Tom20, Tom40, or Tom70 clearly decreased the import of A*β* [40]. The import into mitochondria was insensitive to the mitochondrial membrane potential dissipater valinomycin, indicating that it is independent of the mitochondrial membrane potential [40].

Immunoelectron microscopy showed a consistent localization pattern of A*β* to the mitochondrial cristae; the integration of A*β* into the inner mitochondrial membrane, site of ETC, is in line with results showing that A*β* may cause inhibition of complex IV [43]. A similar labelling pattern was obtained with immunoelectron microscopic analysis of human brain biopsies [40].

Wang et al. [44] investigated the effect of APP and A*β* on mitochondrial dynamics in neurons. Confocal and electron microscopic analysis demonstrated that about 40% of neurons overexpressing wild type APP and more than 80% of cells overexpressing mutant APP displayed alterations in mitochondrial morphology and distribution [44]. Specifically, mitochondria exhibited a fragmented structure and an abnormal distribution accumulating around the perinuclear area [44]. These mitochondrial changes were abolished by treatment with *β*-site APP-cleaving enzyme inhibitor IV [44]. From a functional perspective, APP overexpression affected mitochondria at multiple levels, including elevating reactive oxygen species (ROS) levels, decreasing mitochondrial membrane potential, and reducing ATP production, and also caused neuronal dysfunction [44]. Photoconvertible fluorescence labelling technique showed that mitochondria in APP-overexpressing cells were able to fuse, but slower than controls. At the molecular level, dynamin-like protein 1 (DLP1) was significantly decreased, as well as OPA1, the major organizer of the mitochondrial inner membrane, required for the maintenance of cristae integrity [44]. Overexpression of DLP1 in these cells rescued the abnormal mitochondrial distribution and differentiation deficiency, but failed to rescue mitochondrial fragmentation and functional parameters [44]. On the other hand, overexpression of OPA1 rescued mitochondrial fragmentation and functional parameters, but failed to restore normal mitochondrial distribution [44]. Overexpression of APP or A*β*-derived diffusible ligand treatment also led to mitochondrial fragmentation and reduced mitochondrial coverage in neuronal processes [44]. Therefore APP overexpression, through A*β* production, may perturb mitochondrial dynamics, impacting mitochondrial function and neuronal function [44]. These findings suggest that abnormal mitochondrial dynamics could be involved in mitochondrial and neuronal dysfunctions in AD patients, according with the decreased number but increased size of mitochondria reported in vulnerable neurons of human AD brain specimens [33].

## 4. Mitochondrial DNA Damage in Alzheimer's Disease

To explain the origin of the bioenergetic deficits in AD, cell depleted from endogenous mtDNA have been repopulated with mitochondria (with their own mtDNA) from AD patients and normal controls (cytoplasmic hybrid cells, or “cybrids”) [45]. This application showed that the enzymatic defects can be transferred to mtDNA-deficient cells, thus implicating mtDNA mutations [46]. AD cybrids showed also overproduction of amyloidogenic A*β* peptides (1–40, 1–42), accumulation of amyloid deposits similar to amyloid plaques seen in AD brains, as well as major vulnerability to apoptosis [47]. The worsening of the bioenergetic impairment occurred in long-term culture [48]. Although not all studies with cybrid cells detected differences between AD patients and controls [49], the majority of these demonstrated similar morphological and biochemical phenotype between cybrid cells and cerebral tissue in sporadic AD, supporting the hypothesis that mtDNA changes might be involved in the mitochondrial impairment of sporadic AD. For a complete discussion, see our recent review [7]. Therefore, it has been speculated that aging-related mtDNA mutations may result in impaired energy production, increased amount of ROS, and cell damage, and subsequently neurodegeneration and AD pathology (see Figure 1).

In AD brains, endothelial cells of vessels with atherosclerotic lesions and nearby perivascular cells have been reported to contain clusters of normal and deleted mitochondrial genomes [50]. Chronic hypoperfusion may be an element involved in the pathogenesis of AD, triggering oxidative stress and mitochondrial dysfunction [51]. Aging and cerebrovascular comorbidity could impair cerebral perfusion, subsequently inducing brain capillary degeneration, and suboptimal delivery of energy substrates to neuronal tissue [52]. Mitochondrial dysfunction, oxidative stress, decreased ATP production and increased calcium entry, abnormal protein synthesis, cell ionic pump deficiency, signal transduction defects, and neurotransmission failure resulting from hypoperfusion may contribute to the progressive cognitive decline characteristic of AD and neurodegeneration [50, 53]. In endothelial and perivascular cells of human AD brain microvessels have been detected clusters of mitochondria-derived lysosomes and necrotic changes. Ultrastructural evaluations with probes for human normal and 5-kB deleted mtDNA showed that in AD brain microvessels, but not in age-matched control brains, were present mtDNA deletions [54]. Immunocytochemical analysis demonstrated that the mitochondrial abnormalities in neurons were associated with increased markers of lipid peroxidation [54]. An hypothetical sequence of events for AD progression may go from oxidative damage (protein nitration, lipid peroxidation, nDNA and mtDNA damage, RNA oxidation) to the formation of preneurofibrillary tangles inducing irreversible neuronal damage [51].

Increased levels of 8-hydroxyguanosine (8-OHG), index of mtDNA damage, have been reported in the hippocampus and cerebral neocortex in AD, but not in the cerebellum [55]. Interestingly, levels of 8-OHG were inversely related to the amount of intracellular oligomeric forms of A*β*, suggesting a complex interplay between ROS and A*β* [56]. MtDNA resulted to have approximately 10-fold higher levels of oxidized bases than nDNA, that guanine is the most vulnerable base to DNA damage, and that multiple oxidized bases are significantly higher in AD brain specimens in comparison to controls [57]. Oxidative DNA damage is repaired either in nuclei and in mitochondria by the DNA base excision repair (BER) process [58]. Mitochondria have an independent BER machinery, characterized by a sequence of polymerase and ligase, whose reduction in functionality has been reported in brains of patients with AD, resulting in elevated levels of unrepaired mtDNA [59].

## 5. Mitochondrial DNA Mutations in AD Brains

An increase of somatic mtDNA rearrangements has been observed in AD brains. The mtDNA “common deletion” has been reported to be elevated about 15-fold in AD brains [60]. Furthermore, the mtDNA A4336G transition was observed more frequently in AD patients [61].

More recently, mtDNA control region (CR) mutations have been reported as more frequent in AD brains than in controls [62]. In particular, two heteroplasmic changes were specific for AD brains (T414C and T477C) [62]. 65% of the AD brains harboured the T414G mutation, whereas this mutation was absent from in all control samples [62]. The mtDNA CR from patients and control brains has been cloned and sequenced. AD brains had an average 63% increase in heteroplasmic mtDNA CR mutations (and 130% increase in patients older than 80 years) [62]. These mutations preferentially altered known mtDNA regulatory elements. The AD brains showed also an average 50% reduction in mtDNA content and in the ND6 complex I transcript, which may likely reduce the mitochondrial oxidative phosphorylation [62].

On the other hand, another study involving a larger number of tissue samples did not identify the T414C mutation in AD brains [63]. Elson et al. sequenced the complete coding regions of 145 autoptic AD brain samples and 128 normal controls, and observed that for both synonymous and nonsilent changes the overall numbers of nucleotide substitutions were the same for the AD and control sequences [64]. Therefore, no surely causative mtDNA mutations have been reported in AD patients.

## 6. Mitochondrial Haplogroups and Alzheimer's Disease

The relatively rare familiar forms of AD are associated with mutation in APP and presenilin genes. The causes of sporadic form of AD, that constitutes the *great majority * of the cases, are still unknown. The aetiology of sporadic AD is multifactorial, involving environmental and genetic factors. The major risk factor in sporadic AD is recognized in the allele *ε*4 of apolipoprotein E (*ApoE4*). 

Polymorphisms in mtDNA may cause differences in the encoded proteins, resulting in changes in respiratory chain activity and increasing free radicals. This may result in a predisposition, for an individual or a population with the same polymorphism, to develop early apoptotic processes, accumulation of mitochondrial damages, and somatic DNA mutations [65]. In mice, mtDNA polymorphism seem to be involved in cognitive functioning [66].

The basal branching structure of mtDNA variation in most parts of the world is now well understood [67]. African haplogroups fall into seven major families (L0, L1, L2, L3, L4, L5, L6). About 85 thousand years ago, probably in the Horn of Africa, the root of haplogroup L3 gave rise to many descendant haplogroups (probably because of some colonization event or local population growth). Non-African mtDNA descend from L3 and belong either to the M or N superclades. In the Indian subcontinent and in Southeast Asia there is the richest basal variation in the three originated by haplogroups M and N, and this suggests a rapid colonization along the southern coast of Asia, about 60 thousand years ago [67]. The expansions northwords occurred later, about 45 thousand years ago. Over 30 subclades of the haplogroup M are present in Asia. Haplogroups A, B, C, D, and X have been found in the Americas, coming mainly from Asia. In Europeans and Near Easterners (who share a rather recent common ancestor) nine different mitochondrial haplogroups have been identified (H, I, J, K, T, U, V, W, X). The variation in the basal European mtDNA haplogroups dates to about 45000 years ago. Complete mtDNA sequencing and the increasing number of samples analyzed allow subdividing haplogroups in smaller groups identifying younger branches on the mtDNA evolution tree. Therefore, subhaplogroups classification is continuously evolving [67].

Specific mitochondrial haplogroups have been linked to longevity [68–70]. Therefore, if they can be associated with longevity, the same or other haplogroups could be involved in neurodegeneration. Haplogroup distribution has been reported to differ between normal controls and patients affected with some neurodegenerative diseases, such as Parkinson's Disease [71]. Because of the sensitivity of mtDNA as a marker for human migration patterns, all studies of mtDNA haplogroup association with disease must pay rigorous attention to the ethnic matching of cases to controls [65].

The identification of a possible role for mitochondrial genomic dysfunction in AD, and at the same time the unsuccessful research for mtDNA mutations in AD patients [64], encouraged to study polymorphisms in mtDNA of AD patients. The different studies obtained contrasting results. Chagnon et al. [72] reported that haplogroups T was underrepresented in AD patients, and that haplogroups J overrepresented. In an Italian sample of subject, instead, haplogroups K and U had a lower frequency in *apolipoprotein * (*Apo*) *E4 * carriers, whereas in control subjects this correlation was not present [73]. Therefore, haplogroups K and U may play a role in neutralizing the effect of the major known AD risk factor *E4 * allele [73]. van der Walt et al. reported that haplogroup U in males was related to a significant increase in risk of developing AD, while in females seemed to be associated to a significant protection [74].

Very recently, Maruszak et al. [75] evaluated the involvement of mitochondrial haplogroups, haplogroup clusters (HV, UK, TJ, IWX) and of two functional mtDNA single nucleotide polymorphism (mtSNPs 4216 and 4917) in the pathogenesis of AD in the Polish population. These authors observed that HV cluster seemed to be significantly associated with the risk of AD, regardless of the ApoE4 status [75]. The same study reported no evidence for the involvement of haplogroup U, K, J, or T in AD risk [75]. Two studies including only neuropathologically proven cases of AD of European descent indicated that mtDNA haplogroups were not associated with AD [64, 76].

A study performed in our laboratory evaluated the frequency of the European mtDNA haplogroups in a clinically well-defined group of 209 unrelated patients and 191 controls, both with clear Tuscan origin, in order to minimize the risk of false associations between gene markers and disease [77]. The frequency of haplogroups H, I, J, K, T, U, V, W, and X was not significantly different between patient and control groups, without significant difference between genders [77]. *ApoE4 * allele was significantly more frequent in patients than in controls, and was not associated with any haplogroup [77]. Our data also excluded any association between mtDNA haplogroups, age of onset and mean survival [77].

## 7. Conclusion

The etiology of AD is complex, and only a minority of cases appears to be primarily genetic. *Changes of the expression of mitochondrial and nuclear genes, encoding parts of cyt c oxidase and NADH dehydrogenase enzyme complexes, may contribute to alterations of oxidative metabolism in AD * [78]. *The majority of cybrid studies demonstrated similar morphological and biochemical phenotype between cybrid cells and cerebral tissue in sporadic AD, supporting the hypothesis that mtDNA changes might be involved in the mitochondrial impairment of sporadic AD*. Although morphological, biochemical, and genetic mitochondrial abnormalities have been clearly reported in AD, the role of the mitochondrial genome and of its haplogroups as a risk factor is still controversial. To date no surely causative mtDNA mutations have been discovered in AD patients. Also studies attempting to identify mtDNA mutations in brains of AD patients obtained controversial results. The mtDNA alterations that cybrid models induce to hypothesize might be due to somatic factors, that is, cronic hypoperfusion and oxidative damage.

MtDNA deletions themselves may contribute to aging, dementia, and AD pathology, but the exact mechanism of that is still unclear. Most likely, the mtDNA do not play a primary role, and, therefore, it should be involved subsequently (see Figure 1). Indeed, the APP “stocked” in the TOM transporters and the altered mitochondrial dynamics seem pivotal, able to cause mitochondrial impairment, respiratory deficiency and oxidative stress.

It will be important to develop a better understanding of the role of oxidative stress and mitochondrial energy metabolism in AD, and its link with the amyloid hypothesis in aging and AD, since it may lead to the development of more effective treatment strategies for this devastating disorder.
